# Assessment practices for dietetic students: An updated systematic review (2017–2024)

**DOI:** 10.1111/1747-0080.70001

**Published:** 2025-03-05

**Authors:** Janica Jamieson, Claire Palermo, Margaret Hay, Simone Gibson

**Affiliations:** ^1^ Edith Cowan University Joondalup Western Australia Australia; ^2^ Monash University Australia

**Keywords:** assessment, dietetics, education, health occupations students, systematic review

## Abstract

**Aim:**

Evaluate assessment practices and outcomes for dietetic students and compare findings with those from a previous systematic review.

**Methods:**

A systematic review was conducted whereby four databases (MEDLINE, Embase, Cumulative Index in Nursing and Allied Health Literature, and Education Resources Information Centre) were searched on 11 October 2023 with terms related to dietetics, students, and assessment. The search was repeated on 8 January 2025 to identify new publications. Eligibility criteria were primary research published after 1 June 2017 reporting at least one assessment method for dietetic students with an assessment‐related outcome. Assessment practices and outcomes were evaluated using Miller's Pyramid, the New World Kirkpatrick's Hierarchy, and the principles of programmatic assessment.

**Results:**

From 5701 search results, 22 were identified, revealing new assessment practices, including entrustable professional activities, e‐portfolios, and programmatic assessment, localised to Australia and Singapore. Compared to publications prior to 2017, a greater proportion conceptualised assessment as part of a system (46% compared to 28%) with a sustained higher prevalence of *does* and *shows* levels of Miller's Pyramid. Evaluation continued to focus on reaction, learning, and behaviour.

**Conclusions:**

Findings indicate a transition towards programmatic approaches to systems of assessment within dietetics, though this shift was not observed globally. Such a shift is crucial for ensuring the profession's agility in responding to modern disruptors and maintaining the delivery of high‐quality education.

## 1 INTRODUCTION

In recent decades, the assessment and certification of health professions students have undergone a paradigm shift, marking a departure from reliance on single assessment tools towards a systematic approach to assessment.^1^ This transition has been driven by the recognition that no single assessment tool can adequately capture the complexity of professional competence.^2^ Traditionally, health professions education, including dietetics, has focused on the psychometric properties of individual assessment tools and methods, particularly reliability and validity.^3^, ^4^, ^5^ Whilst this approach has contributed to the refinement of specific assessment tools and methods, challenges have emerged concerning the fragmentation of competence, reductionism of human attributes to quantitative data, the tendency to disregard subjectivity, and the de‐prioritisation of feedback, which are counterproductive to learning.^4^

Emerging from this paradigmatic shift is the concept of ‘programmatic assessment’, which embodies the principles that progression decisions should be informed by broad sampling of a student's performance using multiple, purposefully selected assessment tools and methods applied across diverse contexts, and involving various assessors.^6^ Programmatic assessment represents a systematic, longitudinal approach where rigorous tools and methods are intentionally used to drive and capture student learning and performance. This approach enables the triangulation of information to inform high‐stakes progression decisions proportional to their impact on the student's educational outcome.^6^, ^7^ Programmatic assessment has been implemented across a spectrum of health profession disciplines, such as medicine, paramedicine, as well as in other fields including veterinary science, teaching and communications.^8^, ^9^, ^10^ Programmatic assessment has been shown to facilitate and enrich feedback and learning within assessment processes, and when supported by sufficient evidence, can effectively inform high‐stakes progression decisions.^11^ Whilst remedial action is normalised and destigmatised, serving to individualise the learning process and guide performance improvements.^12^ Although promising, programmatic assessment has been challenged by over‐assessment, resistant workplace‐based supervisors, constraints related to time and finances for design and implementation, design choices dilemmas, difficulties integrating formative assessment, and the persistence or perceived reliance on summative assessment.^8^, ^11^, ^13^, ^14^, ^15^ Despite these obstacles, interest in programmatic assessment continues to grow,^1^ evidenced by the increasing number of implementation examples and evaluation studies,^11^, ^16^, ^17^ Yet, it remains unclear whether this growing interest has translated into tangible shifts towards programmatic assessment in the preparation and certification of dietetic students.

Interest in programmatic assessment has intensified with the emergence of generative artificial intelligence, leading to the proposal of a systematic assessment approach to mitigate challenges posed for higher education.^18^ The rapid and widespread availability of artificial intelligence has profoundly disrupted the landscape of higher education with few, if any, disciplines unaffected.^19^ In health professions education, pedagogical practices are already being shaped by artificial intelligence, with educators actively exploring its potential for teaching and learning,^20^ including dietetics.^21^ Whilst artificial intelligence presents numerous opportunities for innovation within education,^22^ it also poses substantial risks to the integrity and security of many traditional assessment tools and methods, particularly non‐invigilated tasks.^19^ Although on‐campus invigilated assessments, such as closed‐book examinations, may circumvent the misuse of artificial intelligence, they often fail to provide comprehensive insights into student learning and may not always promote equity or student wellbeing.^18^, ^23^ Consequently, there is a pressing need to adopt a systematic and integrative approach that prioritises continuous, multifaceted assessments over singular uncoordinated high‐stake assessments.^18^ Employing a variety of assessment tools and methods across contexts enables educators to triangulate data, leading to a more holistic and trustworthy understanding of student learning and capabilities.^18^, ^23^ This approach mitigates the risks associated with unethical artificial intelligence use and also promotes deeper learning by encouraging students to engage with content in diverse and meaningful ways.^11^, ^18^ Additionally, it provides an opportunity to teach students how to use artificial intelligence ethically and effectively, equipping them with skills necessary for the future workforce. A shift towards a systematic assessment approach for the dietetic profession is essential for preserving the integrity and relevance of education in an increasingly artificial intelligence‐enabled world.

In a systematic review of published literature conducted in 2017, we found that assessment practices for dietetic students predominantly focused on the development and evaluation of single assessment tools, often applied in isolation.^3^ In highlighting the gap between current assessment practices and best practice, we advocated for the dietetics profession to “…move beyond describing the psychometric properties of single tools and instead design programs of assessment that purposefully combine…data to provide maximal student feedback whilst enabling credible and defensible assessment decisions”.^3^ ^(p.290)^ Since then, the emergence of artificial intelligence has accelerated and intensified the need to transform assessment approaches. Considering these recent disruptions, we recognised the importance of revisiting and updating the systematic review to consider whether the dietetics profession has moved towards evidence‐based practice in assessment. The aim of this systematic review was twofold: (i) to identify and evaluate assessment practices and outcomes for dietetic students published since 2017, and then (ii) to compare these findings with those of the earlier 2017 review. By providing this analysis, our research aims to support the dietetics profession in transitioning towards new, adaptive assessment approaches that better prepare students for the evolving landscape of dietetics, ensuring their readiness to navigate an artificial intelligence‐enabled world.

## 2 METHODS

The systematic review has been reported in accordance with the Preferred Reporting Items for Systematic Review and Meta‐Analyses guidelines.^3^ Ethics approval was not sought. The review was registered with the Open Science Framework (https://osf.io/kj3hg/). With the assistance of a health sciences librarian, the electronic databases of MEDLINE (OVID and EBSCOhost), Embase (Ovid and Elsevier), Cumulative Index in Nursing and Allied Health Literature (EBSCOhost), and the Education Resources Information Centre Plus (ProQuest) were searched on 11 October 2023 with a repeated search conducted on 8 January 2025 to identify new publications. As the previous systematic review searched up until 31 May 2017, the present review searched from 1 June 2017. Where possible, the search date was adjusted in the database. If the day and month could not be adjusted within the database, then the year was set to 2017, and studies were manually screened at full‐text review. The same search strings used in the original systematic review were applied (Figure S1). Publications were then imported into EndNote X9,^24^ and duplicates removed. Reference lists of the full text publications were hand searched.

The review eligibility criteria were publications where students pursued a qualification in dietetics. Studies involving a mix of students from different disciplines were eligible if data specific to dietetic students could be isolated. Publications had to describe at least one assessment method and report at least one outcome measure relating to the assessment as defined by the New World Kirkpatrick's Model (*reaction*, *learning*, *behaviour* and *results*), described below. Outcome measures may have included perception and satisfaction of the assessment, assessment scores or changes in assessment scores, comparison of assessment scores to other variables, and student or assessor behaviour change. No limits were placed on study setting or language. Both published and unpublished primary research were eligible, encompassing quantitative, qualitative, and mixed methods approaches. Abstracts and theses were eligible for inclusion.

Study selection and data extraction were repeated as outlined in the original systematic review using Covidence.^25^ Title and abstract screening were independently completed by three reviewers. The full texts were then retrieved and independently evaluated against the eligibility criteria by three reviewers. Any disagreements amongst the reviewers were discussed and resolved through consensus. Additional duplicates were identified and removed in Covidence.^25^

Data extraction was conducted using the same Microsoft Excel^26^ worksheet as in the original review with the following headings: publication year, location, funding source, study aim and design, study population, type and purpose of assessment, and results. For qualitative studies, data in the form of verbatim quotes and themes were extracted from the results, findings, and discussion sections of publications. Abstracts were also reviewed to identify additional pertinent data. Quantitative data was also extracted verbatim from the source material. The data extraction was conducted by one reviewer whilst another reviewer verified the data for accuracy. Any discrepancies that arose during data extraction were resolved through discussion.

The original review applied the Medical Education Research Study Quality Instrument^27^ to evaluate methodological quality and education outcomes. The Medical Education Research Study Quality Instrument tool has since been modified using a Delphi technique with an expert panel to improve the appraisal quality.^28^ The modified version was used to critically appraise quantitative and mixed methods publications (maximum score 100). The Critical Appraisal Skills Programme checklist^29^ was used for qualitative studies. One reviewer applied the Medical Education Research Study Quality Instrument and Critical Appraisal Skills Programme checklist which was verified by a second reviewer. Differences were resolved through discussion.

Study outcomes were evaluated using the five levels of Miller's Pyramid which are *knows*, *knows how*, *shows*, *does* and *is*.^30^ *Knows* represents foundational knowledge and *knows how* the understanding of how to apply such knowledge. *Shows* involves the demonstration of problem‐solving and reasoning abilities to apply knowledge. Application of knowledge and skills within a professional practice setting occurs at the *does* level with *is* representing professional identity.^30^ The New World Kirkpatrick's Model,^31^ rather than the modified Kirkpatrick's Hierarchy, was applied to evaluate study outcomes as it is a modernised version with the same four‐levels of outcomes, these being *reaction, learning, behaviour*, and *results*.^31^ The New World version aligns with outcomes levels used in the original review as Level 1 (participation) is captured in *reaction*, Level 2a (attitudes and perceptions) and Level 2b (knowledge and skills) are given in *learning*, and Level 3 (behaviour change) is consistent with *behaviour*, and *results* is presented within Level 4a (organisation practice) and Level 4b (patient benefits).

The reliability and validity coefficients for assessment were interpreted using the following criteria: values less than 0.5 were considered poor, values between 0.5 and 0.75 were moderate, values from 0.75 to 0.9 were good, and values greater than 0.9 were excellent.^32^ The validity of the assessment was classified as either demonstrated or not demonstrated. The programmatic assessment framework, developed and applied in the original systematic review, was applied to evaluate study outcomes.^2^ One reviewer evaluated study outcomes for each publication which was then independently verified by a second reviewer. Discrepancies were resolved through discussion. In alignment with the updated systematic review research question, data from publications were not synthesised as in the original review to evaluate practices and outcomes of dietetic assessment methods, rather data were summarised across a range of characteristics to enable comparison with the original review.

As researchers, we each bring experience as health profession educators, with J.J., C.P., and S.G. specifically being and working within dietetics. Whilst these experiences have driven our interest in the research questions, they may also influence our perceptions and judgements about the assessment practices for dietetic students. We have all recently conducted research and published in the field of dietetic student preparation and have co‐authored several publications included in this review. These close connections to the research questions and data were managed by adherence to the systematic review protocol and two researchers independently undertaking data collection and analysis. We actively engaged in discussion to challenge each other's interpretations of the data, utilising the study protocol as a guide for such conversations and decisions.

## 3 RESULTS

**FIGURE 1:**
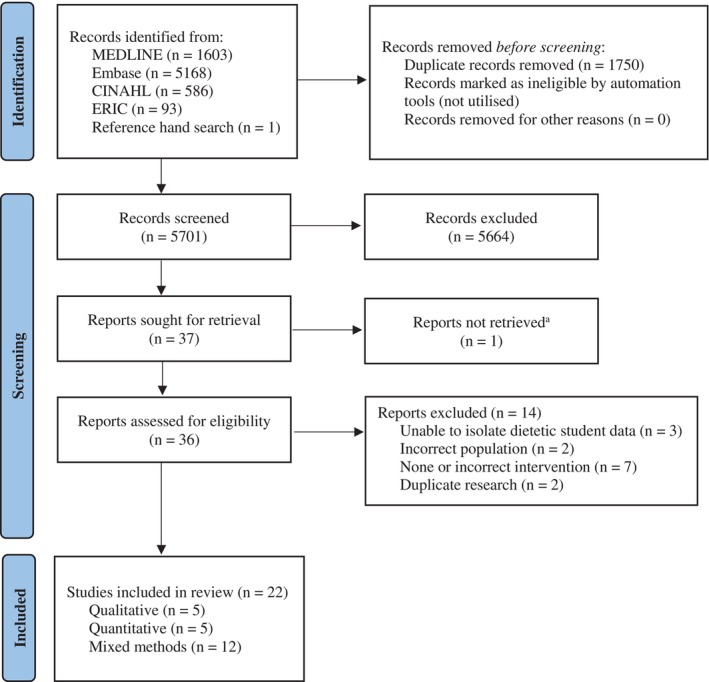
Search and selection process for systematic review into assessment practices for dietetic trainees (search conducted on 11 October 2023 and repeated on 8 January 2025). ^a^Record could not be located by the researchers or health sciences librarian.

The search identified 5701 publications after removal of duplicates, with 22 included for data synthesis following application of the eligibility criteria (Figure 1). Eighteen were journal articles and four were conference proceedings,^33^, ^34^, ^35^, ^36^ with no studies repeated across included publications. English language abstracts were available for all publications.

The highest frequency of publication was observed in 2021 (*n* = 5),^37^, ^38^, ^39^, ^40^, ^41^ noting that only part of the year was included for 2017 and 2025. Thirteen studies were from Australia^35^, ^37^, ^38^, ^39^, ^40^, ^42^, ^43^, ^44^, ^45^, ^46^, ^47^, ^48^ and one of these was a collaboration with New Zealand,^49^ four were from the United States,^33^, ^36^, ^41^, ^50^ and one each from Argentina,^34^ Singapore,^51^ United Kingdom,^52^ Turkey,^53^ and Japan.^54^

The most frequent assessment types were entrustable professional activities (*n* = 6, developed and applied within three settings)^37^, ^38^, ^42^, ^43^, ^46^, ^51^ which are key tasks or responsibilities that students must perform independently to demonstrate competence. This was followed by Objective Structure Clinical Examinations or practical examinations (*n* = 5),^41^, ^44^, ^49^, ^50^, ^52^ and e‐portfolios (*n* = 4, applied within one dietetic training programme),^37^, ^38^, ^42^, ^43^ followed by programmatic assessment (*n* = 2),^39^, ^40^ written tasks (n = 2),^35^, ^45^ tests or questionnaires (*n* = 2),^33^, ^34^ an individual oral interview (*n* = 2),^47^, ^48^ placement artefacts (*n* = 1),^48^ a interprofessional teamwork appraisal (*n* = 1),^53^ and a placement performance appraisal (*n* = 1).^54^ One study considered a variety of formative assessment tasks.^36^ Singular assessments were situated within a system of assessment^38^, ^42^, ^43^, ^45^ for 10 publications from Australia and Singapore.^37^, ^38^, ^39^, ^40^, ^42^, ^43^, ^45^, ^46^, ^48^, ^51^

Assessments occurred in the university (*n* = 10)^33^, ^35^, ^36^, ^41^, ^44^, ^47^, ^49^, ^50^, ^52^, ^53^ and placement (*n* = 9)^37^, ^38^, ^40^, ^42^, ^43^, ^45^, ^46^, ^48^, ^51^, ^54^ settings with one publication spanning both.^39^ One publication did not report the setting.^34^ The most frequent type of assessments within the university setting were Objective Structured Clinical Examination or practical examination (*n* = 5) which were located in the United States,^41^, ^50^ the United Kingdom,^52^ Australia^9^ and an Australian and New Zealand collaboration.^49^ Entrustable professional activities (*n* = 6)^37^, ^38^, ^42^, ^43^, ^46^, ^51^ and e‐portfolios (*n* = 4)^37^, ^38^, ^42^, ^43^ were the most frequent assessment types within the work‐based learning setting, derived from two Australian institutions and Singapore.

For those publications reporting an assessor type (*n* = 16), the most common were university‐based (*n* = 11),^35^, ^40^, ^41^, ^44^, ^45^, ^46^, ^47^, ^49^, ^50^, ^52^ followed by student self‐assessment (*n* = 5)^54^ and work‐based assessors which were described in the same publications as students.^37^, ^38^, ^42^, ^43^ One study described a combination of both university‐based and work‐based assessors (*n* = 1)^48^ and another study, which described programmatic assessment, used a progression committee (*n* = 1).^39^

University‐based assessments evaluated communication and counselling skills (*n* = 6),^41^, ^44^, ^47^, ^49^, ^50^, ^52^ dietetic‐specific skills such as diet assessment and portion estimation (*n* = 2),^35^, ^36^ cultural knowledge, attitudes, and behaviours (*n* = 1),^33^ and interprofessional teamwork (*n* = 1).^53^ In the work‐based learning setting, competency was evaluated broadly (*n* = 4)^37^, ^40^, ^46^, ^51^ and also specifically for clinical (*n* = 3),^38^, ^42^, ^54^ preventative health (*n* = 2),^43^, ^48^ and food service (*n* = 1).^45^ Publications which evaluated competence used entrustable professional activities (*n* = 6),^37^, ^38^, ^42^, ^43^, ^46^, ^51^ e‐portfolios (*n* = 4),^37^, ^38^, ^42^, ^43^ programmatic assessment (*n* = 2),^39^, ^40^ written tasks or artefacts (*n* = 2)^45^, ^48^ and a performance appraisal tool.^54^ Publications are summarised in Table 1.

**TABLE 1:** Assessment practices and outcomes for dietetic students: description of studies (updated review 1 June 2017 to 8 January 2025).

Author(s) year country	Assessment type	Assessment purpose and description	Assessment setting	Assessors	Miller's pyramid^a^	The New World Kirkpatrick's model^b^
Andrade^33^ 2019 United States	Questionnaire	41 item questionnaire measuring cultural knowledge (*n* = 12), attitudes (*n* = 6), behaviours (*n* = 8), activities undertaken by student (*n* = 4) and demographic data (*n* = 2).	NR	NR	Knows	Level 1
Barker et al.^44^ 2024 Australia	Online individual interview	Oral interview to assess readiness for clinical placements. Students received patient case 48‐hours prior, including medical, social, and dietary data, and prompted to prepare a non‐assessed nutrition care plan. Interview was 20‐minutes and had six questions focussed on problem identification, diet recommendations, and communication skills.	Curriculum	University	Shows	Level 3
Bramley et al.^34^ 2021 Australia	e‐portfolio with EPAs	Three separate e‐portfolios for clinical, food service management, and community and public health nutrition placements (>110 days total) with embedded student and WBA appraisals. At end of placement, students are expected to be assessed at unsupervised practice for competencies. 35 EPAs under six categories (data collection, interview skills, diagnosis/problem identification, nutrition management, time management, professional behaviour) scored using a modified Bondy/Entrustment 4‐point scale (1 = not yet demonstrated, 2 = below expected standard, 3 = expected standard, 4 = above expected standard). EPAs embedded within e‐portfolio.	Workplace (clinical, food service, community)	Student (self) WBA	Does	Level 1
Bramley et al.^35^ 2021 Australia	e‐portfolio with EPAs	37 EPAs for clinical practice listed under six categories (data collection, interview skills, diagnosis/problem identification, nutrition management, time management, professional behaviour) and milestones embedded within e‐portfolio with student and WBA appraisals completed twice during placement (mid‐way and end), in addition to weekly supervisor sheet and other learning artefacts. E‐portfolio provided opportunities for formative and summative feedback. Students and WBAs receive a separate 60‐minute orientation to the e‐portfolio. E‐portfolio included written instructions and description of “passing” student expectations. Student performance scored on 4‐point scale (1 = observing, 2 = highly assisted, 3 = minimally assistance, 4 = work ready).	Workplace (clinical)	Student (self) WBA	Does	Level 1
Bramley et al.^40^ 2022 Australia	e‐portfolio with EPAs	40 EPAs for community and public health practice listed under seven categories (needs assessment, planning, implementation, evaluation, dissemination of results, cultural competency, professional behaviour) embedded within e‐portfolio that includes student and WBA appraisals. Appraisals completed twice during placement (mid‐way and end), in addition to weekly goal sheet and other learning artefacts. Four‐point entrustment scale, with additional ‘not assessed’ item, evaluated student performance, reflecting supervisor input for student to perform the EPA.	Workplace (community)	Student (self) WBA	Does	Level 1
Bramley et al.^39^ 2023 Australia	e‐portfolio with EPAs	As described in Bramley et al. 2021.	Workplace (clinical)	Student (self) WBA	Does	Level 2
Dart et al.^36^ 2021 Australia	Programmatic assessment	Programmatic assessment with 40 separate and varied assessment moments and seven evaluation time‐points across 2‐year postgraduate course, which includes 22 weeks placement. High‐stakes progression decisions undertaken by a progress committee with identification of ‘at‐risk’ students and remediation.	Curriculum Workplace	Progress committee	Does	Level 3
Guastavino et al.^49^ 2017 Argentina	Test	Food Intake Assessment Skills which test portion and nutritional content estimation using computer images of hospital meals.	Curriculum	NR	Knows	Level 2
Jamieson et al.^37^ 2021 Australia	Programmatic assessment	Programmatic assessment for placement (20 weeks) component of 2‐year Master. Student learning captured as low‐stakes assessments and collated to inform high‐stakes progression decision. Students and WBA participate in student‐led competency development meetings to support progression and remediation.	Workplace	University	Does	Level 3
Kleve et al.^45^ 2024 Australia	Placement artefact(s) and individual interview	Placement artefact(s) are a team‐based assessment, negotiated with the placement organisation to reflect authentic practice and assess collective competence. Artefact format and quantity varied (e.g., report, literature review, audio‐visual presentation). A rubric assessed outcome value, feedback incorporation, engagement, methods, credibility, literacy, audience appropriateness, and socio‐ecological focus using a scale (0–5 points). The individual interview was a structured, behaviour‐based assessment with ten questions evaluating students' ability to independently plan, implement, and evaluation nutrition programs (scored as yes, no, not applicable), theory application, and communication skills, scored out of 30.	Workplace	University and workplace	Does	Level 3
Kolcu Mi et al.^52^ 2025 Turkey	Teamwork assessment tool	Adapted Simulation‐based interprofessional Teamwork Assessment Tool (SITAT) into the Turkish language (SITAT‐TR) which assesses individual performance within interprofessional teams across 16 items, each scored on a 4‐point scale (total score 16 points).	Curriculum	NR	Does	Level 3
Lawrence et al.^41^ 2023 Australia	Assignment	Dietary assessment assignment requiring completion of food record, diet analyse, and report writing. Assignment modified to included two optional formative assessments with automated marking and feedback in addition to summative assessment.	Curriculum	University	Shows	Level 1
Palermo et al.^42^ 2018 Australia	Oral case/client	Four different oral assessment that required students to demonstrate nutrition counselling/communication skills with a client or case to determine readiness for clinical placement. Assessment duration was 20–45‐min in length. Students received a 1–2 h briefing and could role play prior to the assessment. Case content scaffolded prior learning on clinical theory and communication skills.	Curriculum	University	Shows	Level 3
Parkin et al.^51^ 2019 United Kingdom	OSCE	Practical assessment with four stations (10 or 15 min each) whereby two were active (communication, application of Model and Process for Nutrition and Dietetics Practice, professionalism) and two passive stations (discriminatory and interpretation skills, application of knowledge) occurring either after or prior to placement. Active stations had simulated patient with script and were assessed by experience examiners using scoring sheet for knowledge and communication skills and verbal questions. Simulated patients and examiners briefed prior to assessment clarify script, standardise responses, and clarifying scoring. Active states undergo moderation. Students develop skills during class in preparation.	Curriculum	University	Shows	Level 2
Porter et al.^43^ 2019 Australia	Placement report	Report produced by student(s) based on activities during food service placement and used to assess application of evidence‐based practice, engagement with stakeholders, and standard of work outputs. Structure, medium, and content varied in response to university, placement site requirements, and student activities. Report is one of the artefacts used to determine student competence, others include WBA feedback, student reflections and self‐appraisal, and work presentation.	Workplace	University	Does	Level 3
Qamar et al.^47^ 2019 United States	Varied	Student offered a variety of formative assessments (case studies/worksheets, blogs/discussion forums, group projects, exams/quizzes, essays/writing projects) to enhance engagement in online advanced nutrition course.	Curriculum	NR	Knows	Level 1
Tomesko et al.^38^ 2021 United States	OSCE	Two formative teaching OSCEs (TOSCE) occurred twice before summative OSCE. Assessed against NFPE items using five‐point scale (novice to competent) with face and content validation. TOSCEs involved reviewing patient chart (5 min), conducting food and nutrition history, nutrition‐focused physical examination, and summarising findings with simulated patient (25 min), followed by debrief with simulated patient, fellow students, and faculty (10 min). Acuity of case increased between first and second TOSCE. In the second, students self‐evaluated with performance recording. Summative OSCE was graded by faculty with no post‐debrief.	Curriculum	University	Shows	Level 1
Tyler et al.^48^ 2021 United States	OSCE	Pairs of students provided with NFPE training packet and undertook formative NFPE OSCE whilst a trained observer recorded performance and provided feedback. Students then completed semester teaching followed by a timed NFPE, using OSCE format, with a simulated patient with two trained observers. Observes were trained through the Academy of Nutrition and Dietetics NFPE workshop. Academy of Nutrition and Dietetics NFPE worksheet used as rubric. Healthy actors verbally reported physical malnutrition when required. Simulated patients trained with written guide and given list of potential student questions. Assessment modified based on student outcomes and feedback. Students required to submit ADIME documentation within 1‐week of OSCE, assessed using rubric developed by faculty.	Curriculum	University	Shows	Level 2
Wright et al.^35^ 2017 Australia	EPAs	Fourteen EPAs: (i) apply knowledge of measurement issues to improve care for individuals, populations or systems, (ii) conduct project planning in public health and community nutrition, (iii) critique and develop nutrition education resources, (iv) design policies for monitoring the level of quality in a nutrition service, (v) develop a business or marketing plan for a new product or service with budget, (vi) develop and present recommendations informed by a community, situation and determinant analysis, (vii) engage in professional communication in the social media, (viii) facilitate a nutrition education session, (ix) facilitate a workshop or discussion group to present gathered evidence, (x) manage medical nutrition therapy for patients with complex nutrition conditions requiring lifestyle change, (xi) manage medical nutrition therapy for patients with non‐complex nutrition conditions in acute care, (xii) present recommendations for practice informed by a literature review, (xiii) provide consultation to staff team and management on food service issues, and (xiv) work collaboratively within teams. Framework for assessment including student and peer written appraisal, portfolio of student performance outputs (nutrition care plans, reports), self‐reflection, preceptor and clinical educator appraisals, and student interview to verify assessment evidence. Entrustment decisions occurred mid‐way and at conclusion of placements with faculty evaluated evidence of student performance.	Workplace	University	Does	Level 1
Wright et al.^46^ 2022 Australia and New Zealand	Practical exam	Assess communication and counselling skills. Student provided with case information (referral letter and food diary) (40 m) followed by on‐line consultation, structured using NCP, with simulated patient (20 minutes). Performance assessed with validated global communication rating scale from medicine.	Curriculum	University	Shows	Level 1
Yamamoto et al.^53^ 2024 Japan	Clinical competence	Perceived Nutrition Care Competencies (PNCC) evaluates competence with 50 questions across: nutritional assessment, diagnosis, supplementation, education, counselling, collaboration with related fields, monitoring, basic competency, medical ethics, and interpersonal relationships. Each question scored on 5‐point Likert scale (1 = difficult to practice, 1 = a little (20–30%) can be practised, 3 = half can be practised, 4 = 60–70% can be practised, 5 = more than 80% ca be practised).	Workplace	Student	Does	Level 3
Zainuldin et al.^50^ 2023 Singapore	EPAs	Four EPAs: (i) conducting nutrition assessment, (ii) identifying and prioritising food‐ and nutrition‐related problems, (iii) planning and implementing nutrition intervention and health promotion, and (iv) monitoring, evaluating and continuing nutrition care. Entrustment for students by the end of training was set at level 3, defined as student allowed to enact EPAs only under reactive/indirect supervision.	Workplace	NR	Does	Level 1

Abbreviations: ADIME, Assessment, Diagnosis, Intervention, Monitoring, Evaluation; EPA, Entrustable Professional Activities; NFPE, Nutrition‐focused physical examination; NR, not reported; OSCE, Objective Structured Clinical Examination; PNCC Perceived Nutrition Care Competencies; SITAT‐TR Simulation‐based interprofessional Teamwork Assessment Tool (Turkish); TOSCE teaching OSCE; WBA work‐based assessor.

^a^ Miller's Pyramid levels: knows, knows how, shows, does, and is.

^b^ The New World Kirkpatrick Model: Level 1 = reaction, Level 2 = learning, Level 3 = behaviour, Level 4 = results.

For the quantitative and mixed methods publications (*n* = 17), mean modified Medical Education Research Study Quality Instrument score was 56.0 ± 8.9 (range 40.0–72.0) from a maximum score of 100. Common study weaknesses were single group cross‐sectional (65%),^33^, ^34^, ^35^, ^36^, ^37^, ^38^, ^41^, ^45^, ^48^, ^52^, ^53^ lack of power calculations reporting (100%), low or no response rate reported (65%),^33^, ^34^, ^37^, ^40^, ^41^, ^42^, ^43^, ^48^, ^50^, ^52^, ^53^ and single institution or multi‐centre without details (94%).^33^, ^34^, ^35^, ^36^, ^38^, ^40^, ^41^, ^42^, ^43^, ^47^, ^48^, ^50^, ^52^, ^53^ Eleven studies (65%) provided detailed participant characteristics^37^, ^38^, ^40^, ^41^, ^42^, ^43^, ^45^, ^47^, ^48^, ^52^, ^54^ and of those studies with insufficient detail (*n* = 6), four were conference abstracts. Six studies reported data on assessment by participant (35%),^33^, ^36^, ^37^, ^38^, ^40^, ^43^ one reported knowledge measurements (6%)^34^ and another reported applied knowledge measurements (6%),^35^ with all other studies reporting objective skill measurements (*n* = 9, 53%).^41^, ^42^, ^45^, ^47^, ^48^, ^50^, ^52^, ^53^, ^54^ Seven studies reported internal structure (41%),^33^, ^34^, ^45^, ^47^, ^50^, ^53^, ^54^ 12 reported content validity (71%)^33^, ^38^, ^40^, ^41^, ^42^, ^43^, ^47^, ^48^, ^50^, ^52^, ^53^, ^54^ and 6 (35%) reported relationships to other variables including comparisons between student and supervisor scoring,^42^ novice and expert performance,^34^ formative and summative assessment outcomes^35^ and placement outcomes.^47^, ^52^ Data analysis was appropriate in all studies except one which presented select student feedback.^41^ Three of the five qualitative studies were considered well designed^39^, ^44^, ^51^ with two applying data collection methods insufficient to answer the research question.^46^, ^49^

When applying the programmatic assessment principles framework, 52% (*n* = 11) described multiple tools within a system of assessment,^37^, ^38^, ^39^, ^40^, ^42^, ^43^, ^45^, ^46^, ^48^ with two describing multiple assessments that were not positioned within a system.^36^, ^41^ Of the 10 studies assessing at the *know* to *shows* levels of Miller's Pyramid, 4 (40%) considered the assessment tool validity.^33^, ^34^, ^44^, ^47^ At the *does* level, 8 of the 12 studies (67%) considered methods for user validity, typically through a description of assessor training.^38^, ^39^, ^40^, ^42^, ^43^, ^45^, ^48^ Seven studies (32%)^38^, ^39^, ^40^, ^42^, ^43^, ^46^, ^48^ considered assessment on a stakes‐based continuum. Assessment provided meaningful feedback and aligned with curriculum objectives in 16 studies (73%)^35^, ^36^, ^37^, ^38^, ^39^, ^40^, ^41^, ^42^, ^43^, ^44^, ^45^, ^46^, ^47^, ^48^, ^50^, ^52^ and expert judgement was considered in 11 studies (50%).^37^, ^38^, ^39^, ^40^, ^42^, ^43^, ^45^, ^46^, ^47^, ^48^ Appraisals are summarised in Table 2.

**TABLE 2:** Assessment practice and outcomes for dietetic students: Critical appraisal of studies (updated review 1 June 2017 to 8 January 2025).

Author(s) Year	Modified MERSQI score	Validity and reliability	Key outcomes	Programmatic assessment criteria					
				Multiple items	Instrument validity	User validity	Stakes‐based	Driving learning	Expert judgement
Andrade^33^ 2019	46.5	Content validity and internal reliability demonstrated (Cronbach 0.86).	‐	N	Y	N/A	N	N	N
Barker et al.^44^ 2024	72	Assessment design validity demonstrated using Kane's Validity Framework.	Majority of students (80.5%) passed interview (score range 36–96%). Oral interview predicted placement outcomes, with a significant difference observed between interview score and placement results (*p* = 0.001). Significant difference in interview scores between students who passed placement (70.6% ± 11.6%) and those who passed with remediation (*p* = 0.04, 64.8% ± 12.3%) or failed (*p* = 0.01, 53% ± 12.5%). Anecdotally, oral interview was less resource‐intensive than objective structured clinical examination.	N	Y	N/A	N	Y	Y
Bramley et al.^34^ 2021	46	‐	WBA preferred EPAs and reported low satisfaction with e‐portfolio, improvement needed for usability. Student accepted e‐portfolio and EPAs; perceived it to assist learning. Overall, preference for EPAs embedded into e‐portfolio with a modified Bondy/Entrustment scale to describe student performance.	Y	N/A	N	N	Y	Y
Bramley et al.^35^ 2021	54	Face validity, feasibility and acceptability demonstrated (>3.75/5 Likert scale)	Students (4.12 ± 0.69) and WBA (4.03 ± 0.68) report positive experience with EPAs and e‐portfolio. Students (4.14 ± 0.73) and WBA (4.02 ± 0.09) agree EPAs within e‐portfolio effective to assess performance.	Y	N/A	Y	Y	Y	Y
Bramley et al.^40^ 2022	53	Content and context validity, feasibility and acceptability demonstrated.	EPAs revised and reduced after unfavourable student and WBA evaluation. Students ambivalent regarding effectiveness of EPAs to assessment performance, favourable evaluation for e‐portfolio. WBAs perceived EPAs effective and accurate in assessment of performance. Students prefer five‐point and WBA prefer four‐point entrustment scale.	Y	NA	Y	Y	Y	Y
Bramley et al.^39^ 2023	64.5	Construct validity demonstrated.	Student self‐assessment, using four‐point entrustment scale, increased longitudinally during placement and were statistically significant for all but four EPAs (EPA30, EPA33, EPA34, EPA35) (*p* < 0.001). WBA assessment, using same four‐point scale, increased longitudinal and were statistically significant for all but four EPAs (EPA30, EPA31, EPA33, EPA35) (*p* < 0.001). No difference between WBA and student EPA rating, using four‐point entrustment scale, at placement end (*p* = 0.03).	Y	NA	Y	Y	Y	Y
Dart et al.^36^ 2021	‐	‐	Programmatic assessment increased assessment decisions confidence, reduced burden, increase assessment value, contributed to early identification and remediation of ‘at risk’ students, and required philosophy and practices shift.	Y	NA	Y	Y	Y	Y
Guastavino et al.^49^ 2017	50.5	Discriminant validity, internal reliability demonstrated (Cronbach's alpha 0.70).	RDs median score (57%) was higher than novice student (39.9%). Differences observed between RD and intermediate student (*p* < 0.000), and intermediate student and novice student (*p* < 0.000).	N	Y	N/A	N	N	N
Jamieson et al.^37^ 2021	53	‐	No difference in supervisor perceptions pre‐post programmatic assessment. Programmatic assessment positively transformed student–WBA relationship, WBA needed to be valued, students were empowered, assessment was fit‐for‐purpose, and WBA required training.	Y	N/A	Y	Y	Y	Y
Kleve et al.^45^ 2024	62.5	‐	Students performed better in teamwork‐based artefact (mean 88.9%, range 61%–100%) compared to individual oral interview (mean 73.4%, range 50%–100%) (*p* < 0.001). No correlation between student performance in individual interview and teamwork artefact, indicating that interview performance does not predict artefact performance.	Y	N/A	Y	Y	Y	Y
Kolcu Mi et al.^52^ 2025	62.5	High content validity demonstrated (item content validity index 0.95–1.00 and scale content validity index 0.98). Internal reliability demonstrated (Cronbach's alpha 0.915). Factor analysis determined (Kaiser‐Meyer‐Olkin 0.940 and Bartlett's test of sphericity *p* < 0.001).	‐	N	N/A	N	N	N	N
Lawrence et al.^41^ 2023	48	‐	44% students completed a formative assessment with greater completion by ‘high achievers’ (50%) compared to ‘low achievers’ (20%). For dietary assessment skills, students completing a formative assessment scored higher on subsequent summative assessment (82% vs. 74%, *p* < 0.05); no difference observed for food record skills. Students rated formative assessments as helpful.	N	N	NA	N	Y	N
Palermo et al.^42^ 2018	‐	‐	Agreement observed across assessor scores (12/16 assessors had >75% agreement) with all instances of disagreement occurring when student performance was rated borderline or fail.	N	Y	NA	N	Y	Y
Parkin et al.^51^ 2019	61.5	‐	Difference in OSCE score observed for students that went on to pass (mean 63.24%, SD 7.94), struggle (mean 58.25%, SD 8382) and fail (mean 57.31%, SD 8.28) placement (*p* < 0.00); independent of OSCE timing. No difference in OSCE mean score between students who failed and struggled placement (58.25 and 57.19, respectively). Of those students who failed one active station (9–10%), 64% went on to pass placement. 46% (22/48) of students requiring additional support during placement passed. Students reported the OSCE was meaningful and fair (92%) and helpful for preparation for practice (82%).	N	N	NA	N	Y	N
Porter et al.^43^ 2019	64	‐	Variation between assessor scores, no consistency in rank and student outcome; lessened when dichotomous categories applied (unsatisfactory/satisfactory). Assessors were influenced by quantity and quality of report, student's written communication ability, and need to situate report relative to other learning artefacts.	Y	N/A	Y	N	Y	Y
Qamar et al.^47^ 2019	40	‐	From a maximum score of nine, group projects and essay/writing ranked lowest (7.43 ± 2.03 and 7.04 ± 2.01, respectively) with case studies/worksheets were highest (3.46 ± 1.43). All students preferred timed‐online exams available over several days.	Y	N	N/A	N	Y	N
Tomesko et al.^38^ 2021	47.5	Face and content validity demonstrated.	86% students passed all competencies and 14% required remediation. All achieved passing grade.	Y	N	N/A	N	Y	N
Tyler et al.^48^ 2021	60.5	Inter‐rater reliability demonstrated (Cohen kappa ranged from 0.608 to 0.810 across three cohorts)	Increase student scores between pre and post NFPE in two of three cohorts (*p* < 0.001).	N	N	NA	N	Y	N
Wright et al.^35^ 2017	‐	‐	EPAs positively received by faculty; perceived to represent professional activities. Anecdotal evidence that student reflections improved with EPAs, enabling entrustment to occur. Students perceived self‐assessment to be easier and reported improved understanding of learning.	Y	NA	N	Y	Y	N
Wright et al.^46^ 2022	‐	‐	Non‐verbal communication skills challenging to assess in telehealth assessment format (76% agreement).	N	N	NA	N	N	N
Yamamoto et al.^53^ 2024	66	Content and construct validity demonstrated. Internal reliability demonstrated (Cronbach 0.873–0.946).	‐	N	N/A	N	N	N	N
Zainuldin et al.^50^ 2023	‐	‐	Entrustment is the indirect supervision with clear progression levels.	NA	NA	N	N	N	N

Abbreviations: EPA, Entrustable Professional Activities; MERSQI, Medical Education Research Study Quality Instrument; NFPE, Nutrition‐focussed Physical Examination; OSCE, Objective Structured Clinical Examination; RD, Registered dietitian; WBA, work‐based assessor.

Assessments targeted 3 of the 5 levels of Miller's Pyramid with 3 studies at the *knows* (17%),^33^, ^34^, ^36^ 7 at the *shows* (32%)^35^, ^41^, ^44^, ^47^, ^49^, ^50^, ^52^ and 12 at the *does* level (55%).^37^, ^38^, ^39^, ^40^, ^42^, ^43^, ^44^, ^45^, ^46^, ^48^, ^51^, ^53^, ^54^ No assessments targeted the *knows how* and *is* levels. At the *does* level, entrustable professional activities were reported in six publications conducted across three different settings (two institutions^37^, ^38^, ^42^, ^43^, ^46^ and one country^46^, ^51^) and three studies either reported on programmatic assessment^39^, ^40^ or situated the assessment tool within a system of assessment.^45^, ^48^

Ten publications focused on outcomes at Level 1 (reaction) of the New World Kirkpatrick's Hierarchy. Of these, publications reported on either the relevance of the assessment to practice^35^, ^36^, ^37^, ^38^, ^49^, ^51^ or the degree of engagement with the assessment.^33^, ^37^, ^41^, ^46^ These publications included outcomes for students, work‐based assessors, and faculty. Assessments were an assignment, e‐portfolios, entrustable professional activities, questionnaire, a practical examination, and a variety of formative assessment, with publications occurring in Australia, the United Kingdom, the United States, and Singapore. Level 2 (learning) considered the acquisition of knowledge, skills, and attitudes and was observed in four publications (18%)^34^, ^42^, ^50^, ^52^ which considered a test, an e‐portfolio with entrustable professional activities, and Objective Structure Clinical Examinations in Australia, Argentina, the United Kingdom, and the United States. Eight publications reported outcomes at Level 3 (behaviour) (36%)^10^, ^39^, ^44^, ^45^, ^47^, ^48^, ^53^, ^54^ with assessment methods including a placement report, oral case examination, individual oral interviews, placement artefacts, interprofessional teamwork, competency performance appraisal, and programmatic assessment, with studies occurring in Australia (*n* = 6), Turkey (*n* = 1) and Japan (*n* = 1).

In the updated systematic review, a higher proportion of publications were conducted in Australia (60%) compared to the original review (16%), whilst the reverse was observed for the United States of America (18% in the update review compared to 57% in the original review). The United Kingdom had a lower proportion of publications in the updated review (5% compared to 19%).

A shift in the types of assessments was evident between the original and updated systematic reviews. Entrustable professional activities, programmatic assessment, and e‐portfolios were observed in the updated review but were absent in the original review. Conversely, two singular performance evaluation tools (9%) were observed in the update review, compared to 59% in the original review. There was a small increase in the proportion of publications exploring Objective Structure Clinical Examinations (18% in the original and 23% in the updated). A comparison of types of assessment is provided in Table 3. Ten studies situated singular assessment methods or tools within a system of assessment in the updated review (45%), compared to the original review where 72% of studies described single assessment methods or tools applied in isolation. Assessments were observed in both the placement and university setting in both reviews. Assessors continued to be from the university, placement setting, and the student, with the updated review also having a single publication that included a progression committee.

**TABLE 3:** Comparison of assessment types between original (publications between 1965 and 31 May 2017) and updated systematic review (publications between 1 June 2017 and 8 January 2025).

Types of assessments	Original review (2017)^3^	Updated review (2025)
Entrustable professional activities	‐	6
Examinations	3	‐
Interviews	‐	2
Objective structured clinical examinations	7	5
Performance evaluation instruments	22	2^a^
Placement artefacts	‐	1
Presentation	1	‐
Programmatic assessment	‐	2
Portfolios	3	4^b^
Quizzes	1	‐
Tests or questionnaire	4	2
Varied, multiple types	‐	1^c^
Written tasks	2	2

^a^ One assessed clinical competence^53^ and one assessed interprofessional teamwork.^52^

^b^ All four publications were for an e‐portfolio at one institution.

^c^ Students were offered multiple formative assessment tasks.^47^

The knowledge and skills assessed continued to represent clinical competence, preventative health, counselling and verbal communication, and dietetic‐specific skills such as portion estimation and diet assessment. Food service, cultural capabilities, and interprofessional teamwork were evident in the updated review. Five publications considered assessment broadly across placement settings (food service, preventative health, clinical) which was not observed in the original review.

Differences between the original and updated review were found in the prevalence of *knows how* (12% and 0%, respectively) and *is* level (7% and 0%, respectively) of Miller's Pyramid. Similar reporting of the other levels was noted, particularly at the *does* level, which had the highest frequency in both reviews (53% and 67%, respectively). The New World Kirkpatrick's Model level 1 (reaction) was consistent with the original review, with 45% falling into this category. Four studies (18%) were at Level 2 (learning) and eight were at Level 3 (behaviour) (36%). No studies achieved Level 4 outcomes (results), which was also observed in the original review. Of the eight studies at Level 3 in the update review, over half (63%) applied a qualitative or predominately qualitative mixed methods methodology.

## 4 DISCUSSION

The updated systematic review revealed that assessment practices within the dietetics profession are transitioning, with a shift away from single, siloed tools and towards systems of assessments. Although this shift was not universally observed, it nonetheless reflects the growing adoption of best practice in student education within the dietetics profession. This trend mirrors broader changes that have occurred within health professions education.^55^

The trend towards publication of systematic approaches to assessment was limited to the Australia context.^37^, ^38^, ^39^, ^40^, ^42^, ^43^, ^44^, ^45^, ^48^ Within Australia and New Zealand, there has been a Community of Practice for dietetic educators since 2015 which has “…focused on conceptualising competency‐based assessment as a system with programmes of assessment…”.^56^ ^(p.458)^ An evaluation of this Community of Practice revealed that it had fostered knowledgeable leaders who were empowered to initiate and drive change within their own institutions and throughout the profession. Members of this community were previously found to adopt a scholarly approach to education practices and engage in collaborative research endeavours,^56^ likely contributing to the prevalence of Australian research observed in the updated review, particularly those studies which conceptualised assessment as a system. This is consistent with broader evidence highlighting that Communities of Practice can facilitate practice change in health professions education through reflection, experiential learning and creating a shared goal.^57^ This underscores the importance of investing in educator capacity‐building and cultivating a culture that encourages innovation and research, as these elements are crucial for driving transformative changes in practices.

The magnitude of transformative change required to shift from isolated, surmountable high‐stakes assessments towards programmatic assessment cannot be underestimated. Given the inherent complexity of assessment interwoven with cultural nuances,^58^, ^59^ this transition is unlikely to be straightforward.^18^ Approaches that scratch the surface or address peripheral issues have been cautioned against, as they are unlikely to yield desired outcomes.^23^ Recent emergent research has provided a framework to guide the successful and sustainable implementation of programmatic assessment.^8^, ^15^, ^58^, ^60^ This research highlights the crucial role of strategic leaders knowledgeable in assessment scholarship, who can drive change and navigate complex processes, relationships, and barriers in implementation.^58^, ^60^ Stakeholders, including faculty, industry, healthcare recipients, and, most importantly, students, will require assessment literacy to create a shared understanding for the intended assessment reform. This collective approach, which fosters shared ideology, will be required to navigate anticipated friction points.^39^, ^58^, ^60^ Traditional roles with inherent implicit authority must evolve to empower students and align with programmatic assessment principles, particularly focusing on feedback for learning.^11^, ^59^, ^60^ Crucially, institutional and accreditation bodies, guided by best practice scholarship, must evolve to lead widespread transformation.^60^ Ultimately, the successful adoption of systems or programmatic assessment will depend on the collective efforts of all education partners to embrace change and align practices.

Generative artificial intelligence is poised to significantly impact healthcare systems and the practices of healthcare professionals,^20^ including dietitians.^61^ As artificial intelligence becomes increasingly integrated into healthcare systems, there is a need for curriculum reform to ensure that students are equipped with the knowledge and skills to comprehend and ethically apply emerging technologies to optimise outcomes for care recipients.^62^ Generative artificial intelligence offers promising opportunities for educational innovation, such as the use of chatbots for the development of communication skills, simulated clients, and personalised learning and real‐time feedback, to name a few early examples.^19^, ^20^, ^21^, ^63^ Yet one of the most significant implications of artificial intelligence is the erosion of assessment integrity and the ensuing challenges this poses for certifying student competence.^19^ With the rapid pace of technological advances, relying on a single assessment to mitigate unethical artificial intelligence use by students is unlikely to be effective.^18^ Instead, there is a need to intentionally design and integrate multiple assessment tools and methods that, when triangulated within and across a system, enable holistic and trustworthy judgements about a student.^18^, ^23^ Rather than being a hinderance, the advent of artificial intelligence provides an opportunity to reform our practices by ethically incorporating artificial intelligence into teaching and assessment, fostering inclusive universal design, emphasising a student‐centred approach, and enhancing feedback for learning.^23^ The observed shift towards systematic assessment approaches within the review is beneficial in light of the opportunities and challenges associated with artificial intelligence.

The updated review demonstrated that the highest attainment according to The New World Kirkpatrick's Model was Level 3 (behaviour), achieved using qualitative, mixed methods, or quantitative research methodology. Increasing application and diversity of qualitative research methodology within health professions education research has been observed.^64^ Scholars are moving beyond traditional experimental post‐positivist methods to gain insights into how interventions are implemented and outcomes achieved.^31^ There is growing application of programme evaluation models such as realist evaluation, theory‐driven evaluation, and RE‐AIM framework within health professions education. These models utilise qualitative or mixed methods to trace intervention activities to outcomes, which facilitates a detailed understanding as to how higher level outcomes are actualised.^31^ The findings of the present updated systematic review suggest that such research approaches have yet to be widely applied to dietetics education research, indicating a gap in current evidence. Bridging this gap may be necessary to determine if education practices in dietetics are indeed leading to the intended outcomes.

As the review relied on published research, it may not capture the full scope of current practices in dietetic education. Research conducted during the review period may have experienced disruptions or delays because of the COVID‐19 pandemic. Education scholarship can entail a lengthy process of design, implementation, data collection, and publication, which can extend over several years.^8^, ^15^ Consequently, there is a potential delay between the adoption of contemporary assessment practices and publication, if they are researched and published at all, which may have resulted in the exclusion in the present review. This limitation highlights the importance of fostering collaboration and scholarship within the dietetic education community to collectively innovate and adapt practices and continue to meet the evolving needs of dietetic education.

This systematic review has revealed a transition within the dietetics profession towards adoption of systematic approaches to assessment, a change critical for addressing the challenges posed by modern disruptors such as artificial intelligence. Whilst not observed universally across international contexts, the shift appears to have been facilitated by educational communities that empower educators to implement and research best practice in health professions scholarship. Sustained efforts to bridge the gap between current practices and best practice will be essential for advancing the field and ensuring the delivery of high‐quality education within dietetics.

## AUTHOR CONTRIBUTIONS

JJ was responsible for all components of the research and manuscript. CP, SG and MH contributed to the research protocol and reviewed the manuscript. JJ, CP, and SG screened the publications. JJ completed the data extraction with review by CP and SG.

## CONFLICT OF INTEREST STATEMENT

The authors report no declarations of interest. The authors alone are responsible for the content and writing of the article.

## Supporting information


**FIGURE S1.** Database search strings and fields for the systematic review on assessment practices and outcomes for dietetic trainees (search conducted on 11 October 2023 and repeated on 8 January 2025).

## Data Availability

Research data are not shared.
