# Supplementary material for: Assessment practices for dietetic students: An updated systematic review (2017–2024)
Source: Nutr Diet. 2025 Mar 5;82(5):467–86. doi: 10.1111/1747-0080.70001 (PMC12583895; doi:10.1111/1747-0080.70001)
Supplement: Supplementary file 1 — FIGURE S1. Database search strings and fields for the systematic review on assessment practices and outcomes for dietetic trainees (search conducted on 11 October 2023 and repeated on 8 January 2025). [file NDI-82-467-s001.docx]

| **MEDLINE** (OVID and EBSCOhost^a^) |
| --- |
| Search string: (MH "Dietetics" OR MH "Nutritionists" OR dietitian* OR dietician* OR dietetic*) AND (MH "Students+" OR student* OR learner* OR intern OR interns OR internship*) AND (assess* OR apprais* OR evaluat* OR examination* OR exam OR exams OR competen*)  Field option: ‘All fields’ |
| **Embase** (Ovid and Elsevier^b^) |
| Search string: (“dietitian” OR dietetic* OR dietitian* OR dietician*) AND (“student” OR student* OR learner* OR intern* OR interns OR internship*) AND (assess* OR apprais* OR evaluat* OR examination* OR exam OR exams OR competen* OR 'competence')  Field option: ‘All fields’ |
| **CINAHL Plus and CINAHL Ultimate^c^** (EBSCOhost) |
| Search string: (MH "Dietitians" OR dietitian* OR dietician* OR dietetic*) AND (MH "Students+" OR student* OR learner* OR intern OR interns OR internship*) AND (assess* OR apprais* OR evaluat* OR examination* OR exam OR exams OR competen*)  Field option: ‘All fields’ |
| **ERIC** (ProQuest) |
| Search string: ("Dietetics" OR dietitian* OR dietician* OR dietetic*) AND ("Students") OR student* OR learner* OR intern OR interns OR internship*) AND (assess* OR apprais* OR evaluat* OR examination* OR exam OR exams OR competen*)  Field option: ‘Anywhere’ |

Supplementary Figure 1. **Database search strings and fields for the systematic review on assessment practices and outcomes for dietetic trainees (search conducted on 11 October 2023 and repeated on 8 January 2025).**

* indicates truncation; “MH” or quotations indicates MeSH term; + indicates the explode option for a MeSH term.

^a^ Institutional access to MEDLINE changed; the 11 October 2023 search used OVID, and the 8 January 2025 search used EBSCOhost.

^b^ Institutional access to Embase changed; the 11 October 2023 search used OVID, and the 8 January 2025 search used Elsivier.

^c^ Institutional access to CINAHL changed; the 11 October 2023 search used CINAHL Plus and the 8 January 2025 search used CINAHL Ultimate.
